# Re-Introduction of West Nile Virus Lineage 1 in Senegal from Europe and Subsequent Circulation in Human and Mosquito Populations between 2012 and 2021

**DOI:** 10.3390/v14122720

**Published:** 2022-12-06

**Authors:** Marie Henriette Dior Ndione, El Hadji Ndiaye, Martin Faye, Moussa Moïse Diagne, Diawo Diallo, Amadou Diallo, Amadou Alpha Sall, Cheikh Loucoubar, Oumar Faye, Mawlouth Diallo, Ousmane Faye, Mamadou Aliou Barry, Gamou Fall

**Affiliations:** 1 Virology Department, Institut Pasteur de Dakar, Dakar 220, Senegal; 2 Zoology Medical Department, Institut Pasteur de Dakar, Dakar 220, Senegal; 3 Epidemiology, Clinical Research and Data Science Department, Institut Pasteur de Dakar, Dakar 220, Senegal

**Keywords:** West Nile virus, lineage 1, arbovirus, human population, mosquito population, Senegal, 4S network, mosquito-based surveillance, phylogenetic analysis

## Abstract

West Nile virus (WNV) is a virus of the Japanese encephalitis antigenic complex and belongs to the family Flaviviridae of the genus flavivirus. The virus can cause infection in humans which in most cases is asymptomatic, however symptomatic cases exist and the disease can be severe causing encephalitis and meningoencephalitis. The virus is maintained in an enzootic cycle involving mosquitoes and birds, humans and other mammals such as horses can be accidental hosts. A mosquito-based arbovirus surveillance system and the sentinel syndromic surveillance network (4S) have been in place since 1988 and 2015 respectively, to better understand the transmission dynamics of arboviruses including WNV in Senegal. Arthropod and human samples have been collected from the field and analysed at Institut Pasteur de Dakar using different methods including RT-PCR, ELISA, plaque reduction neutralization test and viral isolation. RT-PCR positive samples have been analysed by Next Generation Sequencing. From 2012 to 2021, 7912 samples have been analysed and WNV positive cases have been detected, 20 human cases (19 IgM and 1 RT-PCR positive cases) and 41 mosquito pools. Phylogenetic analyzes of the sequences of complete genomes obtained showed the circulation of lineage 1a, with all these recent strains from Senegal identical to each other and very close to strains isolated from horse in France in 2015, Italy and Spain. Our data showed lineage 1a endemicity in Senegal as previously described, with circulation of WNV in humans and mosquitoes. Phylogenetic analyzes carried out with the genome sequences obtained also revealed exchanges of WNV strains between Europe and Senegal which could be possible via migratory birds. The surveillance systems that have enabled the detection of WNV in humans and arthropods should be extended to animals in a one-health approach to better prepare for global health threats.

## 1. Introduction

West Nile virus (WNV) is a widespread arbovirus in the world, circulating in all continents except Antarctica [1]. First isolated in 1937 in Uganda [2] from a febrile woman, the virus is a member of the Japanese encephalitis virus (JEV) serocomplex and belongs to the genus *Flavivirus* of the *Flaviviridae* family [2]. A particular characteristic of WNV is its great genetic diversity with at least 9 different lineages circulating in the world [3]. Only lineages 1, 2, 7 and 8 are circulating in Africa. The lineage 7 was recently classified as a separate virus, the Koutango virus by the International Committee on Taxonomy of Viruses [4]. Lineages 1 (the most widespread lineage) and 2 (present in Africa and Europe) are considered the major WNV lineages and are responsible of all WNV outbreaks described in the World [5,6,7,8,9,10]. 

WNV is naturally maintained in an enzootic cycle between birds and mosquitoes [11]. Migratory birds play important role, as they are involved in the long distance viral spread [12,13]. WNV has a broad host range and can be transmitted from mosquitoes to several mammalian species including humans and horses which are considered as accidental hosts [14]. 

The majority of WNV infections (80%) in humans is asymptomatic and infected cases do not necessarily need medical consultancy [15,16,17]. Symptomatic infections are generally characterized by fever, headache, nausea, vomiting, diarrhea, chills, maculopapular rash, joint and muscle pain. The disease can be complicated by severe neurological disorders such as encephalitis or meningoencephalitis (muscle weakness, tremors, paralysis, cognitive impairment and acute flaccid paralysis) [12,18]. However, only about 1% of symptomatic cases present these severe forms that could occasionally lead to death [15,17,19]. 

Before 1990, WNV disease appeared only sporadically and the risk was considered minor for humans. First cases of severe WNV fever were reported between 1994 and 1996 during epidemics in Algeria and Romania where neurological complications were reported in several cases [1]. Indeed, during this epidemic in Romania, more than 500 clinical cases with a severity rate of 10% were reported [20]. Between 1996 and 1999, three major outbreaks of WNV occurred in southern Romania and the Volga Delta in southern Russia, all of which involved hundreds of severe neurological cases and deaths [1,21]. The virus then spread to Europe and epidemics with neurological forms occured recently thereafter in Israel in 2000 with a case fatality rate of 8.4% [22] and in Russia in 2001 [23]. More recently, severe cases of WNV were detected in Greece in 2010, in Italy between 2008 and 2014 [9,24,25], in Hungary and Austria between 2008 and 2009 [26] and in Serbia in 2012 [27].

In America the virus first emerged in 1999 in the United States [7] and has since spread accross America causing nearly 24,656 neurological cases and 2330 deaths in the United States. Currently, WNV is the main cause of encephalitis in the United States [28].

In Africa, the virus seems to have a minor impact. However this may be due to a lack of appropriate surveillance systems as epidemics with neurological cases were detected in Sudan in 2002 [29], in Tunisia in 2003 and 2012 [30,31] and in South Africa between 2008 and 2009 [32]. In Senegal, no outbreak linked to the virus has been documented. However, the virus was detected from mosquitoes, birds, horses and humans [33,34,35,36,37,38,39,40,41]. Regarding humans, sporadic cases with anti-WNV antibodies were found in the population until the 1990s [39,40,41] and two human acute cases were notified for WNV in Dakar, with strains isolated in 1970 and 1991 [41]. Since these years, no additional WNV human cases linked to WNV was documented. To compensate for this lack of informations on WNV and others arboviruses and hemorraghic fevers, many programs were set up to follow the appearance/emergence of sporadic cases or epidemics due to these pathogens. Indeed, since 2011, the Institut Pasteur de Dakar (IPD) has set up in collaboration with the Ministry of Health a countrywide surveillance network named “4S” (Syndromic Sentinel Surveillance in Senegal) focused on influenza viruses and other respiratory viruses [42]. Since 2015, because of an increasing number of febrile cases not related to respiratory viruses, arbovirus and bacteria detection have been included in this surveillance system [43]. Furthermore, this human surveillance is coupled with a mosquito-based arbovirus surveillance system, which has also been in place for a long time and permitted the isolation and identification of many viruses including WNV, from mosquitoes in Barkedji and Kedougou areas [44]. Here we describe WNV in human and mosquito populations in Senegal between 2012 and 2021 with phylogenetic studies showing the lineages circulating. 

## 2. Materials and Methods 

### 2.1. Human Surveillance

#### 2.1.1. Presentation of the Syndromic Sentinel Surveillance Network in Senegal (4S Network) 

Human serum samples were collected through the Syndromic sentinel surveillance network in Senegal (4S network). The 4S network based on syndromic approach is a partnership between Ministry of Health (MoH), the WHO country office and the IPD which hosts the National Influenza Center and the WHO collaborating center for Arboviruses and hemorhagic fever viruses (CRORA). This program was implemented since 2011 [42] to strengthen national capacities for the detection of pathogens responsible for infectious diseases [42]. Then in 2015 the 4S program was extended to monitoring arboviruses and hemorrhagic fever viruses [42].

The 4S sites were selected on the basis of criteria used by the MoH. A checklist criteria was developed based on the WHO recommended attributes for sentinel site selection including, feasibility, representativeness and the availability of data to enable disease burden estimate [42]. Overall, the program includes 25 sentinel sites spread over the 14 regions of Senegal. The epidemiological survey is based on an individual questionnaire put in place for suspected cases. 

#### 2.1.2. Human Sample Collection 

Suspected arboviral infection case was defined as fever and at least two of the following symptoms: headaches, myalgia, arthralgia, skin rash, orbital pain, hemorrhagic manifestations (purpura, epistaxis, gingivorrhagia, metrorrhagia). For each arboviral disease suspected case, a dry tube venous sample is taken and sent to the arbovirus reference laboratory at IPD. All collected samples were tested by differential arboviral RT-PCR and ELISA for the detection of viral genome and IgM antibodies for WNV and other arboviruses [43]. 

### 2.2. Mosquito Collection

Mosquitoes were collected as part of the viro-entomological surveillance of arboviruses, conducted by Institut Pasteur de Dakar since many years. Indeed, we used in this study moquitoes collected from June to December each year, between 2012 and 2020 in the Barkédji area already described in previous papers [44,45]. Indeed, the Barkédji area (15°17′ N, 14°52′ W) located in the Sahelian biogeographic domain, is characterized by a shrub vegetation, hot dry climate, and a short rainy season from June to October, with annual rainfall less than 500 mm. Several temporary pools remain wet for 7 to 8 mo each year. A network of temporary ponds of different sizes is flooded by the first rains. Small ponds are flooded and drained after each rain. Large ponds are flooded at the beginning of the rainy season, remain inundated for a long period and are covered by hydrophytes. These ponds are the main source of water for herders and their livestock in this period. These ponds are also the natural habitats of many vertebrate species (birds, reptiles and rodents) and mosquito vectors of arboviruses. Mosquitoes were collected using a backpack aspirator [46], CDC light traps [47], and indoor residual spaying [48] and frozen in liquid nitrogen. The traps were placed on the edge of 18 ponds and in 22 villages around Barkédji area and mosquitoes were collected monthly in the villages and every two weeks in the ponds. 

We also used mosquitoes collected in 2017 during a dengue outbreak investigation in the Matam region (15°39′ N, 13°15′ W) which is 200 km from Barkédji. The traps used was the same as listed above.

Mosquitoes were later morphologically identified to the species level using morphological keys [49,50] and pooled by species, sex, and date, maximum of 89 and average of 9.8 mosquitoes per pool. 

### 2.3. Serological Diagnostic

Blood samples collected from patients were centrifuged and stored at −20 °C until laboratory assays. Serum samples were tested by immunoglobulin M (IgM) antigen- capture enzyme-linked immunosorbant assay (ELISA) for WNV and other arboviruses using in house methods developed at the CRORA at IPD [51]. Briefly, we coated 96-well microtiter plates with a monoclonal IgM capture antibody (goat anti-human IgM; KPL) in carbonate bicarbonate buffer (pH 9.6) and incubated overnight at 4 °C. After washing the plate 3 times with phosphate-buffered saline plus 0.05% tween, we added heat-inactivated (56 °C, 30 min) human serum samples and controls (all diluted 1:100 in phosphate-buffered saline plus 0.05% tween and 1% milk powder) in duplicate into plate wells and incubated at 37 °C for 1 h. We washed wells 3 times; added ZIKV, DENV, WNV, or YFV antigens into plate wells; and incubated the plate for 1 h. After 3 washes, we added ZIKV-, DENV-, WNV-, or YFV-specific immune ascites from mice to each well and incubated for 1 h at 37 °C. After 3 washes, we added peroxidase-labeled antibody specific to mouse IgG for 1 h at 37 °C. Last, we added a tetramethylbenzidine substrate to the IgM conjugate complex and stopped the color reaction using a sulfuric acid solution. Serum samples were considered positive if the optical density at 450 nm was >0.20 above the negative serum sample average and the ratio between the sample and the negative control was >2.

Due to cross-reactions occuring among species of the *Flavivirus* genus, WNV IgM positive samples were also tested by plaque reduction neutralization tests (PRNTs), also an in-house method developed at the CRORA for confirmation. Briefly, Serial 2-fold dilutions (1/10 to 1/320) of inactivated serum samples were prepared using Liebovitz (L15) medium supplemented with 3% foetal bovine serum (FBS), 1% penicillin streptomycin and 0.05% fungizon) as a dilution medium. Then a test dose dilution (DT) was prepared from the virus stock to obtain 10^3^ PFU/mL (i.e., 30–50 PFU per well). From the DT, the dilution range of the virus (d50, d70, d90, d95, d99) which will make it possible to determine the percentage inhibition of the cytopathic effect of the virus by a serum has been determined. In 96-well plates, the diluted sera and DT were added to the corresponding wells and to the wells reserved for the dilution range of the virus the different doses (DT, d50, d70, d90, d95, d99) and dilution medium were added. The plates are then incubated for 1 h at 37 °C. After 1 h, cell suspension with a concentration of 10^6^ cells/mL was added, followed 4h later by the overlay medium (0.6% Carboxy Methyl Cellulose in dilution medium). Plates were incubated for 4 days at 37 °C and later stained with amido black. The results obtained are interpreted using the viral dilution range and virus neutralizing serum samples such as D90 were considered to have neutralizing antibodies (paper submitted) against WNV. 

### 2.4. Molecular Diagnostic

Extraction of viral RNA from human serum samples and mosquito pools was performed with the QIAamp viral RNA mini kit (Qiagen, Heiden, Germany) according to manufacturer’s instructions. For the detection of viral RNA, we used a consensus WNV real-time RT-PCR assay recently developed and included in the differential diagnosis of arboviruses in our laboratory [52]. The real-time PCR assays were performed using the Quantitect Probe RT-PCR Kit (Qiagen, Heiden, Germany) according to manufacturer’s instructions, in a 96-well plate under the following conditions: 50 °C for 15 min, 95 °C for 15min followed by 40 cycles of 95 °C for 15 s and 60 °C for 1 min, in ABI 7500 thermocycler (Applied Biosystem).

### 2.5. Virus Isolation and Identification

Mosquito pools were homogenized in 3 mL of L-15 medium (Gibco BRL, GrandIsland, NY, USA) supplemented with 20% fetal bovine serum (Gibco) as described previously [44] and centrifugated to perform a filtration using a 1ml syringe (Artsana, Como, Italy) and a 0.20 μm filters (Sartorius, Göttingen, Germany). Mosquito monolayer C6/36 cells were infected with the filtered supernatant and incubated for 7–8 days for cytopathic effect detection. The presence of virus was detected by indirect immunofluorescence assay (IFA) using in-house hyper immune mouse ascites fluids directed to individual or groups of more than 70 African arboviruses (flaviviruses, bunyaviruses, orbiviruses and alphaviruses) done as described previously [53]. Viral identification was later done by complement fixation and seroneutralization tests. Molecular tools were used to confirm this identification [52].

### 2.6. Viral Genome Sequencing

To obtain the complete genomes from WNV RT-PCR positive samples, we used the high-throughput sequencing technique with the Miseq device (Illumina, San Diego, CA, USA). The samples were prepared for sequencing following a protocol adapted from that proposed by Allander and colleagues [54]. Briefly, a depletion of the ribosomal RNA of the host was made followed by the synthesis of the complementary DNA and the random amplification of the DNA fragments by the SISPA method (Sequence-independent, single-primer amplification). Then the libraries were prepared with the Nextera XT Library Prep kit (24 samples), according to the manufacturer’s recommendations. The libraries thus obtained were each identified by indexes (Nextera XT index kit V2, Illumina) then pooled at the same concentration. Whole-genome sequencing was performed with paired-end reads using the Illumina MiSeq reagent kit v2 (300 cycles) on an Illumina MiSeq instrument. To generate the consensus genomes, we used the fully open-source EDGE Bioinformatics software [55].

### 2.7. Phylogenetic Analyses 

The assembled sequences were manually curated using the Unipro UGENE software (http://ugene.net/download.html, accessed on 5 May 2022) [56] and analysed with the online Basic Local Alignment Search (BLAST) program (https://blast.ncbi.nlm.nih.gov/Blast.cgi, accessed on 5 May 2022) to assess the most neighbouring sequence previously available on GenBank (www.ncbi.nlm.nih.gov/genbank/, accessed on 5 May 2022). 129 complete WNV sequences previously available on GenBank and representing the current genetic diversity of WNV were used. Multiple alignments were performed using the Muscle method [57] implemented in the UGENE program. The best-fitting nucleotide substitution model for our dataset was assessed using the ModelFinder program [58] implemented on the IQ-TREE web-server [59], with a discrete Gamma distribution (+G) for 4 categories. The maximum likelihood (ML) tree was then inferred using FastTree v2.1.7 (http://www.microbesonline.org/fasttree, accessed on 5 May 2022) [60] with the 16 complete sequences of old strains from Senegal isolated between 1989 and 2003 already available and 5 complete sequences of new strains isolated between 2012 and 2018 and the best-fitting nucleotide substitution model to our sequence dataset. The ML tree was generated for 5000 replications and nodes were supported by the Shimodaira-Hasegawa test (SH-like) values. The topology was visualized by FigTree v.1.4.2 (http://tree.bio.ed.ac.uk/software/figtree/, accessed on 5 May 2022) [61]. Only SH-like values ≥ 0.8 were shown on the tree and dengue virus was used as outgroup. 

The Bayesian phylogenetic analysis was performed using a general time-reversible model with gamma-distributed rate variation for 4 rate categories (GTR+G4), as selected by Akaike’s information criterion (AICc) in ModelFinder program [58]. The evolutionary analysis was conducted assuming a strict Gamma clock and GMRF Bayesian Skyride coalescent tree prior. Two independent Markov chain Monte Carlo (MCMC) runs with up to 100 million generations were performed using BEAST v1.8.4 (http://beast.bio.ed.ac.uk, accessed on 5 May 2022) and sampling every 100 thousands to ensure the convergence of estimates [62]. Log files were visualized using Tracer [63] to ensure convergence during MCMC by reaching effective sample sizes (ESS) greater than 200. The posterior distribution of trees obtained from the BEAST analysis was also used to obtain the Bayesian maximum clade credibility (MCC) tree for these sequences generated by TreeAnnotator v.2.3.2 [64] (from 100 million) after a 10% burn-in and the topology was visualized by FigTree v.1.4.2 (http://tree.bio.ed.ac.uk/software/figtree/, accessed on 5 May 2022) [61].

## 3. Results

### 3.1. Human Samples

#### 3.1.1. WNV Detection

Among patients enrolled in the 4S between 2012 and 2021, 7912 were arboviral suspected infections (Table 1). Among these serum samples, 20 were found positive for WNV (19 IgM and 1 RT-PCR positive samples) (Table 2). The RT-PCR positive serum samples was detected in 2018 with a Ct value of 31 (corresponding to 3293 copy of RNA/mL). This patient was a 33 year old female who lives in the Matam region, located in the North, and presented fever, headache and retro-orbitary pain symptoms. 

The PRNT titers of the IgM confirmed cases ranged from 1/10 to 1/320 (Table 2). A patient who was sampled one month after its confirmation showed the increase in antibody titer from 80 to 160. These analyzes show an overall percent of WNV circulation in humans in Senegal of 0.24%, varying between 0.077 and 0.78% from 2015 to 2021. 

#### 3.1.2. Distribution of WNV Positive Cases by Age and Sex 

**Figure 1:**
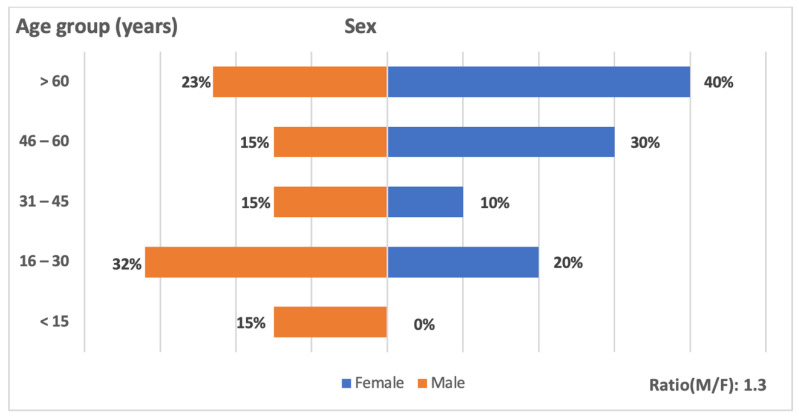
Distribution of WNV cases in Senegal by sex and different age groups, between 2016 and 2021.

Among the positives cases, we obtained 12 men and 7 women (Table 2) and the sex ratio M/F was 1.3 (Figure 1). The mean age of WNV confirmed patients was 39.35 (range: 3 months–77 years). The 16–30 age group is the most affected with seven cases, and there have only been two cases in people over the age of 65.

#### 3.1.3. Spatio-Temporal Distibution of WNV Positive Cases

**Figure 2:**
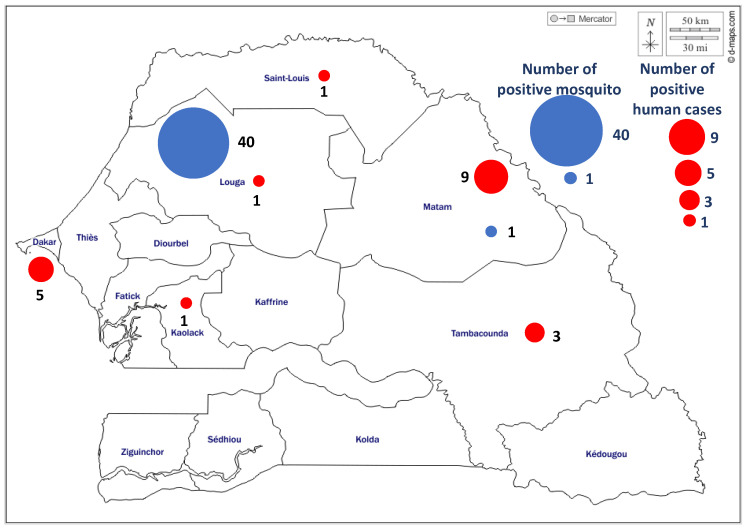
Geographical distribution of human and mosquito cases of WNV in Senegal from 2016 to 2021. Red dots show regions where WNV positive human cases were isolated and blue dots where WNV positive mosquitoes were isolated. Human cases are found according to this map mainly in the north, the west and the south east of Senegal, and mosquito cases mainly in the north.

**Figure 3:**
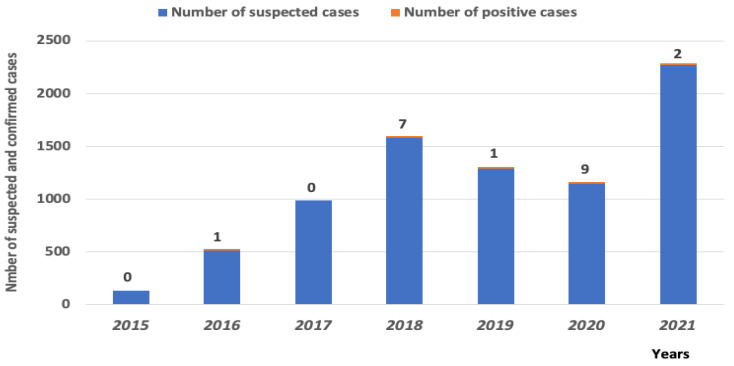
Number of WNV-positive human cases relative to the total number of suspected individuals.

These different cases were distributed in six regions of Senegal, localized in the north Saint-Louis region (1 case), the Northest Matam region (8 cases), in the capital city, Dakar region (5 cases), in the center Louga (1 case) and Kaolack (1 case) regions and the southeast Tambacounda region (3 cases) (Figure 2). More positive cases were found in the northern half of the country with 78.94% of the WNV positive cases. The geographic distribution of positive cases between 2012 and 2021 showed interestingly WNV emergence in new area of the country where the virus has not been previously isolated, in Kaolack (center of Senegal). The positive cases were detected for all years during September to January periods, which cover the raining season of the country. The WNV confirmed serum samples were detected in 2016 (1), 2018 (6), 2019 (1), 2020 (9) and 2021 (2) (Table 2) with the peaks observed every two years, in 2016, 2018 and 2020 (Figure 3).

#### 3.1.4. Symptoms Associated to WNV Disease

**Figure 4:**
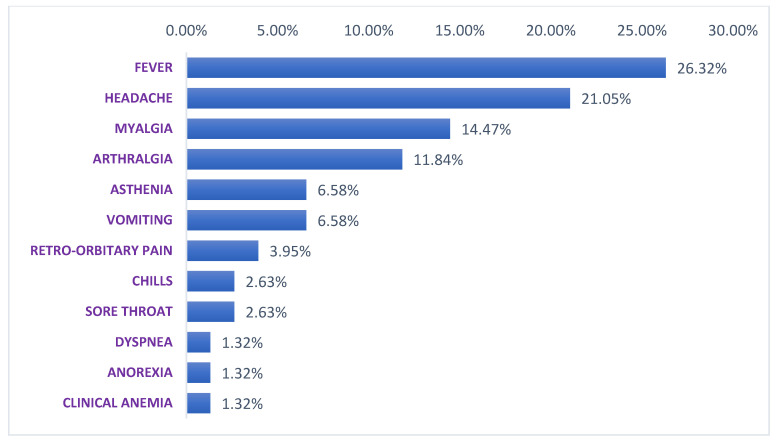
Clinical signs presented by people infected with WNV in Senegal from 2016 to 2021.

These WNV positive patients presented on the day of their blood sampling temperature varying between 36 °C to 39.9 °C (Table 2). Positive patients present common signs which are fever for all patients, headaches (78.94%), myalgia (57.89%), arthralgia (42.10%), asthenia (26.31%), vomiting (26.31%) and some particular signs that are, chills (10.52%), sore throat (10.52%), retro-orbitary pain (10.52%), dyspnea (5.26%), anorexia (5.26%) and clinical anemia (5.26%) (Figure 4). All patients have recovered and no severe form nor death was recorded.

### 3.2. Mosquito Samples

A total of 13,009 mosquito pools mainly corresponding to *Culex (Cx.), Aedes (Ae), Anopheles (An), Mansonia (Ma) and Mimomyia* genus were analysed. Among these mosquito pools, these following mosquito species have been identified: *Cx. neavei, Cx. perfuscus, Cx. quinquefasciatus, Cx. ethiopicus, Cx. titraniorhynchus, Cx. antennatus, Cx. poicilipes, Cx. bitaeniorhynchus, Cx. trigripes, Cx. nebulosus, Cx. decens, Cx. annulioris, Ae. vexans, Ae. aegypti, Ae. dalzieli, Ae. ochraceus, Ae. sudanensis, Ae. argenteopunctatus, Ae. fowleri, Ae. furcifer, Ae. minutes, Ae. squamosus, Ae. unilineatus, Ae. metallicus, Ae. luteocephalus, Ae. hirsutus, Ae. mcintoshi, An. Gambiae, An. Pharoensis, An. rufipes, An. ziemanni, Ma. uniformis, Ma. Africana, Mimomyia splendens, Mimomyia plumose, Mimomyia mymomyiamorfis.*

Overall, 41 mosquito pools were positive for WNV. Indeed, in Barkédji, WNV strains were isolated mainly from *Culex* genus, particularly, *Cx. neavei, Cx. poicilipes, Cx. antennatus and Cx. tritaeniorhynchus* (Table 3). One WNV strain was isolated in Matam from *Cx neavei*. These virus isolations occured from 2012 to 2017. As for human positive cases, WNV positive mosquitoes were detected every year during September to January periods which cover the raining season of the country.

### 3.3. Phylogenetic Analyses of WNV Strains Isolated in Senegal

**Figure 5:**
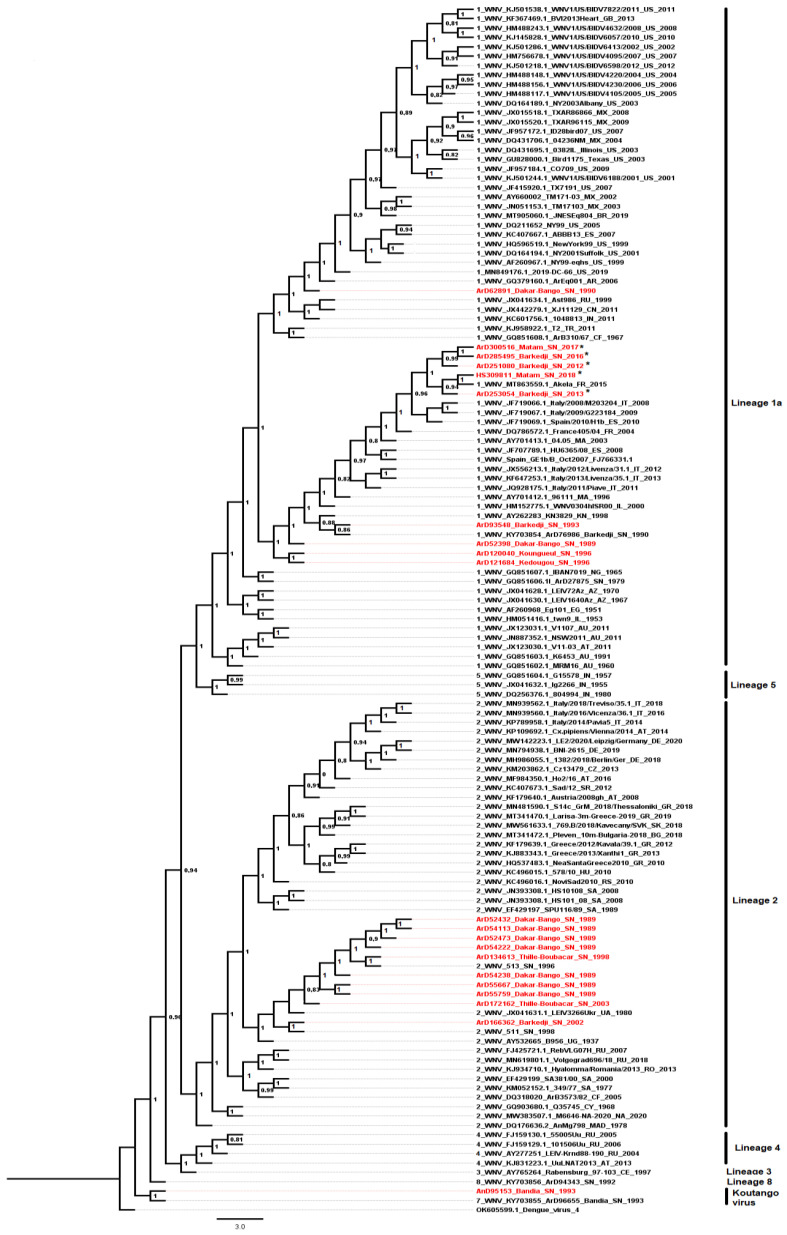
Maximum Likelihood (ML) tree based on complete polyprotein sequences of West Nile virus isolates circulating in Senegal from 1989 to 2018. The tree is midpoint-rooted, nodes are labeled with local support values computed using the Shimodaira-Hasegawa (SH) test for 5000 bootstrap replications. The new characterized WNV isolates from Senegal are color-coded in red in this tree. The most recent strains isolated from a human in 2018 (HS309811_Matam_SN_2018*) and from mosquito pools collected in 2017 (ArD300516_Matam_SN_2017*), 2016 (ArD285495_Barkedji_SN_2016*), 2013 (ArD253054_Barkedji_SN_2013*), 2012 (ArD251080_Barkedji_SN_2012*), respectively, are pointed with the asterisk sign (*). These recent characterized Senegalese WNV isolates are belonging to the lineage 1a.

The phylogenetic analysis revealed the circulation of three WNV lineages (1, 2 and 8) in Senegal as previously reported [19] and showed that the new characterized Senegalese isolates from 2012 to 2018 including four mosquito isolates and one human isolate (Table 4) belong to the lineage 1a (Figure 5).

The MCC tree was scaled to time (years) to the most recent common ancestor (MRCA) with its corresponding 95% highest posterior density (HPD) interval. The newly characterized WNV sequences from Senegal diverged from the MRCA in the late 19th/early 20th century (95% HPD: 1881–1905) and clustered with sequences belonging to lineage 1a which showed multiple introductions into Europe and other New World countries. 

**Figure 6:**
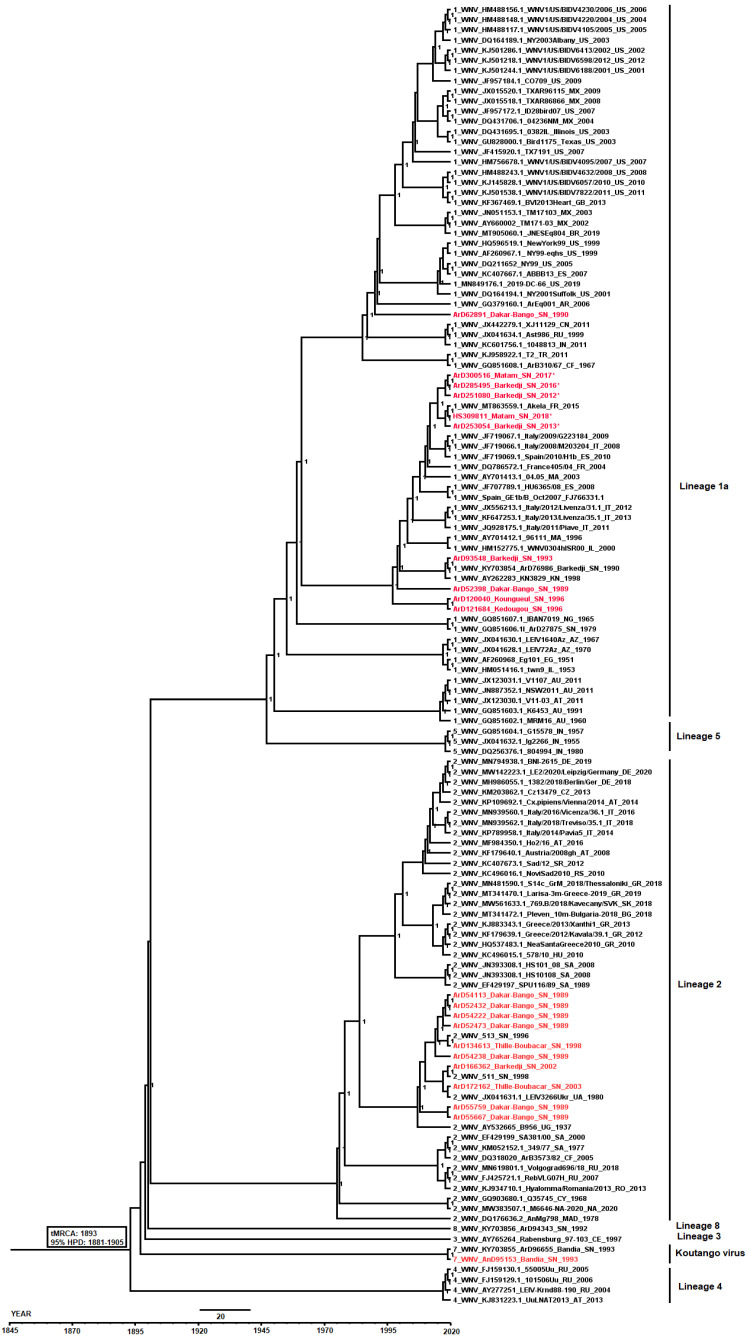
Bayesian maximum clade credibility tree estimating the phylogenetic relationships of West Nile virus including recent sequences from Senegal between 1989 and 2018. Tree nodes were supported by posterior probability values. The Senegalese West Nile virus sequences are colored by in red and the most recent strains are pointed with the asterisk sign (*) for visual clarity. For each sequence, the two-letter code representing a country of isolation is included in the sequence label. Branches are scaled in years before 2020. The most recent strains from Senegal (*) belonged to the lineage 1a and clustered with sequences from Europe. A recent introduction of the lineage 1a into Senegal from Europe was identified.

The lineage 1a exhibited a separate phylogenetic clade that first emerged in Senegal around the early 1990s, spread to Europe during the 2000s and was re-introduced to Senegal from Italy and Spain from the 2010s. The most recent Senegalese sequences, including the human strain isolated in 2018, belonging to the lineage 1a, were linked to an isolate from equine collected in France in 2015 (MT863559) (Figure 6).

## 4. Discussion

Here we describe for the first time the detection of several WNV human cases in Senegal, associated with a strong detection of the virus in the mosquito vector. Indeed, the improvement of arbovirus surveillance in Senegal with the implementation of the 4S system and new diagnostic tools made it possible to highlight the circulation of many arboviruses including dengue, Crimean-Congo hemorrhagic fever, Rift Valley fever and WNV viruses in humans as well as in arthropods [43,44,65,66]. Before the implementation of the 4S network for arboviruses surveillance, WNV detection in humans was limited to two cases detected in Dakar in 1970 and 1991 [41]. Entomological surveillance, as also shown in our study, detected regularly the virus in the center of the country [38,44]. In addition, seroprevalence studies conducted in Senegal, showed high exposition of humans [33,39,40,41], which suggested high endemicity of the virus at least in the center of the country. Few recent studies has shown the presence of the virus in humans in Senegal and this is probably due to the lack of appropriate surveillance systems for WNV detection. After the implementation of the 4S surveillance system, we were able to report 20 human cases of WNV disease between 2016 and 2021 in Senegal thus confirming the circulation of this virus in the human population in Senegal.

Infected persons detected in our study presented minor symptoms and no severe case with encephalitis or meningoencephalitis or death was observed. Indeed, patients came for consultation as early as possible within the first five days after the onset of fever and/or other symptoms, and were therefore able to benefit from treatment very early. This shows the impact of the 4S program in the monitoring of arboviruses, which aims to provide early care for patients. Similarly, various studies have shown that patients over 65 years old are more susceptible to mortality following WNV infection as well as patients with comorbidities [67,68,69]. During our study period, only two cases with similar ages (68 and 77 years old) were detected and the patients had no known comorbidities, which could partly explain the absence of severe cases and deaths in Senegal between 2015 and 2021. 

We obtained more cases in men than in women like some previous studies [70], showing that men are more at risk than women for WNV infection. However, conflicting data exist showed that women are more affected than men [67]. Larger seroprevalence studies covering the whole country could make it possible to better settle this question in Senegal. Our study revealed also that young people are more affected, consistent with previous studies describing that young people had greater risk of developing symptoms [67].

We have during our study detected mostly WNV specific IgM antibodies in suspected patients, showing a recent infection. Only one blood sample was positive by RT-PCR. This failure in detecting viral RNA in blood patients has already been described and could limit genetic characterization of WNV strains circulating in humans. Indeed, it has been suggested than blood sample is not optimal specimen for RNA detection and WNV RNA can be detected in the urine of infected patients during the acute phase of the infection [71,72]. In a large proportion of patients, viral RNA was detected in urine for more longer time than in plasma [71]. Considering these results, urine samples should be taken from individuals suspected of WNV infection to improve detection. 

We also detected more cases in the north-central part of the country compared to the central-south part. This results confirmed previous studies conducted in Senegal that mostly detected WNV strains or specific antibodies in birds, horses and mosquitoes in the north of the country [33,34,35,36,37,38], where the ornithologic park of Djoudj is located. In this park, there are birds known to be reservoirs of WNV including migratory birds from Europe that overwintered in Africa [34]. In the south part of Senegal WNV was more found in Tambacounda region housing another park named Niokolo-koba, from many animals, birds and mosquitoes. Indeed, previous studies carried-out in this part of the country in 1988, 1990 and 1991 had shown anti-WNV antibodies in human population as well as the presence of the virus in mosquitoes [37,38,41,42]. These data suggest that the north-central part seems more favorable to the transmission of WNV and the presence of zoologic parks with birds also seems to constitute a risk factor even in the southern part where the WNV transmission is lower. In Senegal, this distribution of WNV more in the north of the country than in the south has also been demonstrated for RVFV, another endemic arbovirus in northern Senegal [34,73,74] which is also transmitted by mosquitoes of the *Culex* genus. Interestingly, we noted a peak in the number of WNV cases every two years between 2015 and 2021 and always towards the end of the rainy season during which temporary ponds fill up and constitute a favorable biotope for many mosquito species mainly from *Culex* or *Aedes* genus [33,38]. Peaks in case numbers were observed in Europe like our study for 2018 and 2020 [75] although the lineages responsible for the outbreaks were different. Modeling studies for predictive models would confirm this trend of every 2 years and better understand the emergence of the virus in Senegal and around the world. If this trend is confirmed, cases will be detected this year 2022, it is therefore necessary to strengthen surveillance in both vector mosquitoes and humans.

During our study, we detected WNV positive *Culex* mosquitoes earlier, between July and November before the occurrence of human cases. This entomological surveillance constitutes an early warning sytem, which allows early detection of the virus in mosquitoes, and triggers the implementation of anti-vectorial measures to prevent the transmission of the virus to the human population [44,76]. *Culex* mosquitoes are reported to be the primary competent vectors of WNV. Indeed, 26 pools of *Cx. neavei* mosquito positive for WNV out of a total of 46 were detected in our study. Indeed this species of wild mosquito has been shown to be competent to transmit WNV lineage 1 and 8 in Senegal and could be involved in the enzootic transmission of the virus between birds and horses [3]. In addition, one WNV positive *Culex quinquefasciatus* pool has been detected. This mosquito species has been shown to be competent to transmit WNV lineage 1 in Senegal and could be involved in the urban transmission cycle of the virus, as this species is know to be associated with human dwellings [3,77]. Vector competence analysis should also be done for the other *Culex* species mosquitoes found positive in our study, i.e., *Cx perfuscus, Cx. poicilipes, Cx. tritaeniorhynchus, Cx. antennatus* and *Cx. ethiopicus* to estimate their implications in WNV transmission in Senegal. 

In our study, five novel complete genome sequences of WNV were characterized to assess the genetic diversity and the current molecular epidemiology in Senegal with recent circulation in humans occuring from 2016 to 2021. Phylogenetic analyses showed that all recent WNV strains isolated during our study period, from human in 2018 and from mosquitoes in 2012, 2013, 2016 and 2017 in Senegal, belong to the lineage 1a. This lineage, linked to many WNV epidemics in the world has a major impact in public health and has been isolated in Senegal from mosquitoes for many years ago [5,8,19]. Older WNV strains isolated from mosquitoes between 1989 and 2003 belong to lineages 1 and 2. These two lineages as well as lineage 8 were previously described as circulating in Senegal [19]. 

The most recent lineage 1a WNV strains isolated from a human and mosquitoes from Senegal are close to each other. These strains clustered with European sequences from birds and equines to which they are closer compared to other lineage 1a isolated from Senegal between 1989 and 1996. Indeed, these recent strains are closer to the strain isolated from horses in France in 2015. Those data clearly exhibit the WNV exchanges between Africa and Europe by the phylogenetic profile of the lineage 1a that first spread to Europe from Senegal since the 1990s and re-introduced back recently from Europe since the 2010s. Migratory birds could explain these strains exchanges between Africa and Europe, as they are involved in the long distance viral spread [12,13,78]. Indeed, in Senegal migratory birds fleeing the cold in Europe between November and May come to stay in the Djoudj park located in the north, characterized by a permanent pond of water, which attracts many bird species. In this park, WNV was detected in resident birds and mosquitoes, which shows a circulation of the virus in this area [34]. Thus migratory birds can introduce new strains both in Senegal and in Europe. The phylogenetic link between sequences from mosquitoes and humans in Senegal and sequences from birds and equines from Europe highlight a probable circulation of the equine-related WNV strains between mosquito vectors with horses and humans as incidental hosts [12] in Europe and in Senegal. Although, no acute WNV infection was reported in Senegalese equine during the last five years, more sero-epidemiological studies need to be conducted to estimate its recent burden in this incidental host’s group [36]. 

WNV epidemics were reported in Europe during our study period, and the phylogenetic analyzes carried out showed, contrary to what happened in Senegal, the circulation of lineage 2 [75,79,80,81]. Indeed, WNV lineage 2 was restricted to Africa until 2004 when it emerged in Hungary and southern Russia and caused outbreaks in wild birds and a few cases of infection in humans [8,81,82,83]. Today, WNV lineage 2 is endemic in many European countries and is responsible for the majority of human WNV infections [79,80,84]. 

For the majority of the WNV cases in Senegal, only IgM antibodies were detected and phylogenetic analyzes could unfortunately not be carried out to identify the responsible lineages. However, the lineage 1a isolated between 2012 and 2018 could be responsible for these cases, especially in Matam where the highest number of WNV cases was detected as well as two strains belonging to this lineage in 2017 and 2018. Interestingly lineage 1a was detected in Barkedji (Louga region, in the center of the country) in mosquitoes from 2012 to 2016, this lineage could also be the source of the human cases detected in this area and in the surrounding regions. 

Although WNV lineages 1, 2 and 8 have been reported in Senegal [19], no major outbreak nor large frequency of WNV infection derived encephalitis cases, has been reported in the country yet. If syndromic surveillance probably played a significant role in case detection, but perhaps also the emergence of this new strain could lead to an increase in symptomatic infections in human population. In the northern part of Senegal, WNV seroprevalence is high [33,39,40,41] while an emergence in areas with low seroprevalence or even naive populations could probably trigger a WNV outbreak. Surveillance should therefore be strengthened and characterization studies to investigate the virulence of this new strain required. The previous identification of competent mosquito vectors for WNV transmission, particularly for the lineage 1 in Senegal [3], and the recent detection of acute human cases point the need to establish an active surveillance at country level based on a One-health approach including multidisplinary teams. This surveillance can help to better understand factors that are currently driving the emergence of acute human cases in Senegal. The new generated sequences could be useful in future studies assessing the global circulation dynamic of WNV [85]. 

## 5. Conclusions

Despite the presence of at least three different lineages of WNV in Senegal, a major epidemic has never been detected. However, an increasing number of sporadic cases of WNV infection have been reported in recent years through our study. This case appearance could be due to the improved surveillance of arboviruses in humans in the country through the 4S program. The phylogenetic characterization of WNV strains isolated from humans and mosquitoes between 2012 and 2017 showed a circulation of lineage 1a in both mosquitoes and humans, with a similar phylogenetic identity of one strain to each other. It is therefore important to monitor the dynamic of this lineage detected during all these last years. Since it is involved in major WNV outbreaks around the world, it could emerge in its severe form or in areas with low WNV seroprevalence and cause an outbreak. A multidisciplinary approach taking into account the entomological, zoological, ornithological and human aspects for the identification of vectors, reservoirs and hosts, could make it possible to better understand the transmission, maintenance and the burden of WNV in the country.

## Figures and Tables

**Table 1:** Number of tested human serum samples per year between 2015 and 2021.

Years	Number of Tested Samples
2015	123
2016	515
2017	980
2018	1579
2019	1293
2020	1149
2021	2273

**Table 2:** This table shows informations about the clinical data (developed clinical signs, age, sex, temperature) of the WNV-positive patients in Senegal 2016–2021, their residence, the date of collection of their serums, and the results of serological tests carried out (ELISA and PRNT).

IPD Number	Center	Reception Date	IgM	Titer /PRNT	Signs	Age	Sex	T°
283397	PS PONT	18/10/2016	POS	10	FEVER, CEPHALEAS, MYALGIAS, VOMITING	37	M	38.9
309809	PS BOKIDIAWE	18/10/2018	POS	40	FEVER, CEPHALEAS, ARTHRALGIAS,	48	M	39
310012	CS COKI	23/10/2018	POS	80	FEVER, CEPHALEAS, MYALGIAS, ARTHRALGIAS, ASTHENIA, DYSPNEA, CHILLS	30	M	39.1
310192	PS BOKIDIAWE	26/10/2018	POS	>320	FEVER, CEPHALEAS, MYALGIAS	21	F	38
310255	PS SAINT LOUIS	30/10/2018	POS	20	FEVER	3 MONTHS	M	38.8
317510	CS HANN MARISTES	13/11/2018	POS	160	FEVER, CEPHALEAS, MYALGIA, ASTHENIA, CHILLS, VOMITING	28	M	39
317575	DAKAR	15/11/2018	POS	80	FEVER	33	M	
323078	PS BOKIDIAWE	25/11/2019	POS	10	FEVER, CEPHALEAS, ARTHRALGIAS, SORE THROAT	21	F	38.9
326040	PS PONT	24/09/2020	POS	80	FEVER, CEPHALEAS, MYALGIAS, ARTHRALGIAS, ANOREXIA	58	M	39.9
327049	PS BOKIDIAWE	15/10/2020	POS	80	FEVER, CEPHALEAS, ARTHRALGIAS, SORE THROAT	NA	F	38.2
328043	PS BOKIDIAWE	22/10/2020	POS	>320	FEVER, CEPHALEAS, ARTHRALGIAS, CLINICAL ANEMIA, VOMITING	8	M	38
329097	DAKAR	02/11/2020	POS	80	FEVER, CEPHALEAS, MYALGIAS	77	F	38
330006	DISTRICT OUEST	03/11/2020	POS	40	FEVER, CEPHALEAS, MYALGIAS, ARTHRALGIAS, ASTHENIA, VOMITING, RETRO-ORBITARY PAIN	58	F	>38
350013	DISTRICT OUEST	06/11/2020	POS	>320	FEVER, CEPHALEAS, MYALGIAS, ARTHRALGIAS, ASTHENIA, RETRO-ORBITARY PAIN	21	M	39.5
363215	PS BOKIDIAWE	10/12/2020	POS	80	FEVER, CEPHALEAS, MYALGIAS	60	F	39.4
363216	PS BOKIDIAWE	10/12/2020	POS	>320	FEVER, CEPHALEAS, MYALGIAS, ASTHENIAS	65	M	38.7
363264	PS OREFONDE	21/12/2020	POS	20	FEVER	19	M	NA
363431	PS PONT	14/01/2021	POS	10	FEVER, CEPHALEAS, MYALGIAS, ARTHRALGIAS, VOMITING	56	F	39.4
377948	GOSSAS	24/11/2021	POS	20	FEVER	68	M	36

**Table 3:** The numbers of mosquito pools tested per year as well as the number of WNV positive pools and the species of mosquitoes from which the virus was detected between 2012 to 2020 are listed in this table.

Year	Number of Mosquito Pools Collected	Number of WNV Lineage 1 Positive Pools	Area of Virus Isolation	Mosquito Species
2012	2185	18	Barkédji	*Cx. quinquefasciatus* (1), *Cx. perfuscus* (1), *Cx. neavei* (9), *Cx. poicilipes* (1), *Cx. tritaeniorhynchus* (1), *Cx. antennatus* (3), *An. rufipes* (1) and *Ae. dalzieli* (1)
2013	2069	14	Barkédji	*Cx. neavei* (13), *Cx. tritaeniorhynchus* (1)
2014	517	0	NA	
2015	722	0	NA	
2016	1214	8	Barkédji	*Cx. neavei* (3), *Cx. poicilipes* (4) and *Cx. ethiopicus* (1)
2017	2761	1	Matam	*Cx. neavei* (1)
2018	727	0	NA	
2019	859	0	NA	
2020	1955	0	NA	

**Table 4:** ID of the new sequenced strains for WNV phylogenetic characterization in Senegal, the hosts from which they were isolated, their years of isolation, the locality of isolation and their Genbank accession numbers have been specified on this table.

Strain	Origin	Year of Isolation	Locality	Genbank Accession Number
ArD52398	Mosquito	1989	Dakar Bango	OP846971
ArD52432	Mosquito	1989	Dakar Bango	OP870453
ArD54113	Mosquito	1989	Dakar Bango	OP870454
ArD52473	Mosquito	1989	Dakar Bango	OP870455
ArD54222	Mosquito	1989	Dakar Bango	OP870456
ArD54238	Mosquito	1989	Dakar Bango	OP846976
ArD55667	Mosquito	1989	Dakar Bango	OP870457
ArD55759	Mosquito	1989	Dakar Bango	OP870458
ArD62891	Mosquito	1990	Dakar Bango	OP846977
AnD95153	Rodent	1993	Bandia	OP846972
ArD93548	Mosquito	1993	Barkédji	OP846973
ArD120040	Mosquito	1996	Koungueul	OP846974
ArD121684	Mosquito	1996	Kédougou	OP846975
ArD134613	Mosquito	1998	Thille Boubacar	OP870459
ArD166362	Mosquito	2002	Barkédji	OP870461
ArD172162	Mosquito	2003	Thille Boubacar	OP870460
ArD251080	Mosquito	2012	Barkédji	OP846978
ArD253054	Mosquito	2013	Barkédji	OP846979
ArD285495	Mosquito	2016	Barkédji	OP846982
ArD300516	Mosquito	2017	Matam	OP846980
HS309811	Human	2018	Matam	OP846981

## Data Availability

Not applicable.
